# Sympatric genome size variation and hybridization of four oak species as determined by flow cytometry genome size variation and hybridization

**DOI:** 10.1002/ece3.7163

**Published:** 2021-01-14

**Authors:** GaoMing Wei, Xuan Li, YanMing Fang

**Affiliations:** ^1^ Key Laboratory of State Forestry and Grassland Administration on Subtropical Forest Biodiversity Conservation Co‐Innovation Center for Sustainable Forestry in Southern China College of Biology and the Environment Nanjing Forestry University Nanjing China; ^2^ School of Physics, and Electronics Henan University Kaifeng China

**Keywords:** flow cytometry, genome size variation, hybridization, *Quercus*

## Abstract

The *Quercus* species serve as a powerful model for studying introgression in relation to species boundaries and adaptive processes. Coexistence of distant relatives, or lack of coexistence of closely relative oak species, introgression may play a role. In the current study, four closely related oak species were found in Zijinshan, China. We generated a comprehensive genome size (GS) database for 120 individuals of four species using flow cytometry‐based approaches. We examined GS variability within and among the species and hybridization events among the four species. The mean GSs of *Q. acutissima*, *Q. variabilis*, *Q. fabri*, and *Q. serrata* var. *brevipetiolata* were estimated to be 1.87, 1.92, 1.97, and 1.97 pg, respectively. The intraspecific and interspecific variations of GS observed among the four oak species indicated adaptation to the environment. Hybridization occurred both within and between the sections. A hybrid offspring was produced from *Q. fabri* and *Q. variabilis*, which belonged to different sections. The GS evolutionary pattern for hybrid species was expansion. Hybridization between the sections may be affected by habitat disturbance. This study increases our understanding of the evolution of GS in *Quercus* and will help establish guidelines for the ecological protection of oak trees.

## Graphical abstract

We examined GS variability within and among the species and present hybridization among four species. It occurred hybridization both within the section and between the sections. A hybrid offspring produced from *Q. fabri* and *Q. variabilis*, which belong to a different section. The pattern of GS evolution for hybrids species is GS expansion. Hybridization between the sections may be affected by habitat disturbance.

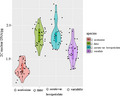

## 1 INTRODUCTION

Nuclear genome size (GS), a critical organismal characteristic and structural component, has high practical and predictive values for studying adaptive or stochastic processes in evolutionary biology (Talla et al., 2017). The GS varies considerably among organisms, with a greater than 2,360‐fold variation existing among different angiosperms (Greilhuber et al., 2006; Leitch et al., 2019). This variation is found within both species and genera (Castro et al., 2013; Denaeghel et al., 2017; Li et al., 2017; Nowicka et al., 2016; Prancl et al., 2014; Qiu et al., 2018). GS evolves in both directions, expanding as well as shrinking. Polyploidization events and transposable element (TE) amplification in plants lead to genome expansion (Hawkins et al., 2008; Kumar & Bennetzen, 1999; Wood et al., 2009), while recombination processes may lead to genome contraction (Devos et al., 2002; Hawkins et al., 2009; Schubert & Vu, 2016). Natural selection exerts a force on the genome; consequently, GS variation is usually related to the living environment, including the altitude, latitude, temperature, and precipitation level (Bennett et al., 2000; Hidalgo et al., 2015; Knight & Ackerly, 2002; Knight et al., 2005; Li et al., 2017; Zhang et al., 2019). GS allows the detection of interspecific hybrids and/or backcrosses (Vit et al., 2014; Yan et al., 2016) and has been widely applied to various plants, such as *Sarcococca* (Denaeghel et al., 2017; Prancl et al., 2014; Tlili et al., 2020).

Flow cytometry (FCM), which is a fast and effective tool to estimate GS, has been successfully applied to ploidy identification, cell cycle analysis, and species identification, including hybrids, rarely occurring cytotypes, and aneuploids (Francis et al., 2008; Hanusova et al., 2014; Vit et al., 2014; Zhang et al., 2019). It has been used successfully in plant genetic variation studies, genetic analyses, and breeding, as well as in studies of reproductive ecology, evolution, and plant system classification (Bilinski et al., 2018; Galbraith, 2004; Sharma et al., 2019; Spaniel et al., 2019). In the 1980s, Galbraith et al. (1983) developed a fast, efficient, and convenient method for isolating plant nuclei, which meant that FCM could be more widely used in botanical research, especially to determine plant GS.

*Quercus* L. is a genus that contains many economically and ecologically important tree species found in the northern hemisphere (Aldrich & Cavender‐Bares, 2011). From an evolutionary point of view, *Quercus* is a good material for studying the species boundaries and adaptive evolution (Porth et al., 2016; Yuan et al., 2018; Manuel et al., 2020; Petersson et al., 2020). *Quercus* is famous for its remarkable natural hybridization, which makes it difficult to identify species owing to the formation of hybrids (Song et al., 2015). Additionally, the frequent hybridization events of oak trees lead to shaping the community assembly and structure, as well as to the evolution of species and the generation of new species (Cannon & Scher, 2017; Wetherbee et al., 2020). Closely related species rarely co‐exist in the same forest land, because hybridization and introgression lead to species merging over time, eliminating their co‐existence (Cavender‐Bares & Pahlich, 2009; Pollock et al., 2015). Even when oaks occur in sympatry, there is significant gene flow. These may include the introgression events that lead to adaptation.

In this study, we found four closely related species of *Quercus* in Zijinshan, China that allowed us to study whether hybridization occurs between sympatric *Quercus*. The four oaks belonged to two sections in the genus *Quercus*. *Quercus acutissima* and *Quercus variabilis* belong to the Section *Cerris*, which is called the Old world clade, while *Quercus fabri*, and *Quercus serrata* var*. brevipetiolata* belong to the Section *Quercus*, which is called the New world clade (Manos et al., 2001). Members of the same sections easily form hybrids (Cottam et al., 1982), and there is an especially high frequency of hybrid formation within the *Quercus* section. We investigated whether hybridization occurs between sections. First, we determine the GSs of the four oak species, and then we determined whether there were variations in the GSs of these oak species. Finally, we analyzed the species hybridization within and between the two sections and determined the evolution of GS in these four oak species.

## 2 MATERIALS AND METHODS

### 2.1 Research site overview and field sampling

**FIGURE 1:**
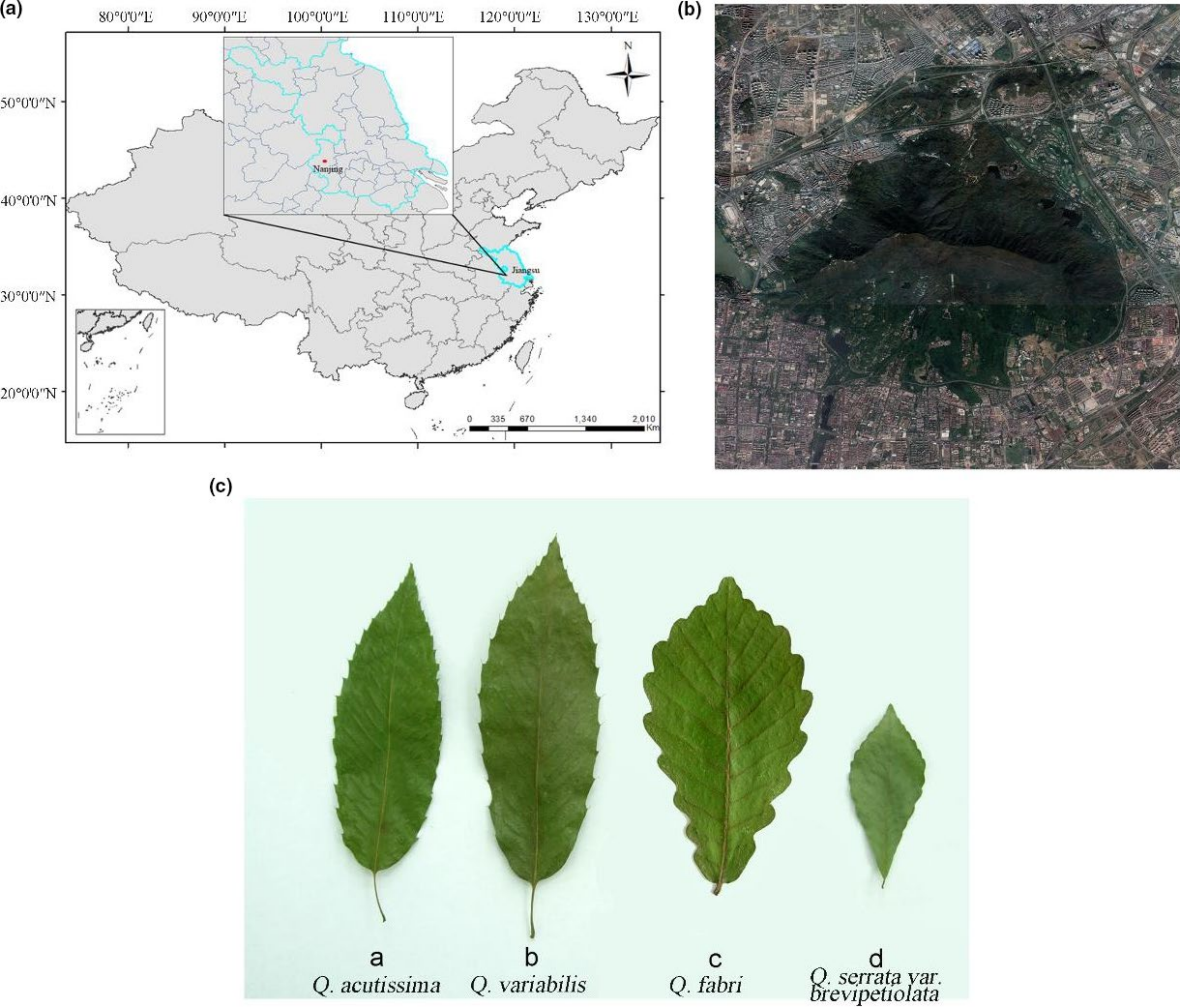
The geographical location of the study area and plant materials (a) Location of the research area in China; (b) Google Earth high‐resolution image of Zijin Mountain; (c) Features of four *Quercus* leaves

A total of 120 samples of *Q. acutissima*, *Q. variabilis*, *Q. fabri*, and *Q. serrata* var. *brevipetiolata* were collected in Zijin Mountains. Zijin Mountains is located to the east of Nanjing, Jiangsu Province (118°48′24″–118°53′04″ E, 32°01′57″–32°06′15″ E) and has an area of 3,008.8 ha, with a forest area of 2,107.6 ha, which has a forest canopy coverage of 0.75–0.80. At present, the vegetation of Zijin Mountains is mainly artificial and natural secondary forests, which are transitional between subtropical evergreen broad‐leaved forests and warm temperate deciduous broad‐leaved forests. The zonal vegetation is a mixture of deciduous evergreen and broad‐leaved forests that is rich in various plant resources. *Q. acutissima* and *Q. variabilis* are important deciduous tree species in this area that are widely distributed and occupy dominant positions. The samples were prepared from the fresh mature leaves of natural wild plants growing in the Zijin Mountains, Nanjing, Jiangsu Province, China, during late October 2011 (Figure 1). The sampling site is a mixed plot with four deciduous oaks, *Q. acutissima* and *Q. variabilis*, *Q. fabri, Q. serrata* var. *glandulifera*. 120 samples of four species of oak including seedlings (DBH ≤ 1), saplings (1 < DBH≤10), and trees (DBH > 10) were collected randomly and evenly in this forest stand.

### 2.2 FCM measurement of the nuclear DNA content

Approximately 200 mg of oak leaves was used for the FCM analysis, and *Petunia hybrida* (2C = 2.85 pg) from the Nanjing Forestry University nursery was included as the internal standard plant (Marie & Brown, 1993). We used the Marie's Buffer (50 mM C_6_H_12_O_6_, 15 mM NaCl, 15 mM KC1, 5 mM EDTA Na_2_, 50 mM Na_3_C_6_H_5_O_7_.2H_2_O, 0.5%(v/v) Tween 20, and 50 mM Hepes, pH 7.2) and 2.2 μl β‐mercaptoethanol should be added to each ml of buffer before use (Favre & Brown, 1996; Marie & Brown, 1993). Owing to the collection of mature leaves, we modified Marie's Buffer to obtain the maximum nuclear yield. The most suitable conventional of β‐mercaptoethanol and Tween 20 for *Q. acutissima*, *Q. variabilis*, *Q. fabri*, and *Q. serrata* var. *brevipetiolata* was 45 mM, 2.5%; 15 mM, 2.0%; 60 mM, 1.0%; 30 mM, 1.0%, respectively (Wei & Fang, 2015). Pipettes were used to immerse 20 mg leaves in 1 ml of optimized Marie's Buffer. The leaves were then quickly chopped in Petri dishes. The dishes were gently shaken so that the chopped leaves and buffer completely mixed. A 25‐μm nylon mesh was used to remove any fragments and large debris. The nuclei were stained with propidium iodide (PI, Fluka, Buchs, Switzerland), and RNase (Fluka) was added to a final concentration of 50 μg/mL. The samples were treated at room temperature for 15 min in a dark environment before being analyzed using a flow cytometer (FACSCalibur, BD USA).

### 2.3 Data analyses

The 2C DNA content was calculated using the following formula:

$$2\text{C DNA}\left(\text{pg}\right)=\frac{\text{sample}(G0)/\text{average fluorescence intensity}(G1)}{\text{Petunia hybrida}(G0)/\text{average fluorescence intensity}(G1)}\times 2.85.$$

The conversion ratio between the DNA mass and Base logarithm was 1 pg = 978 Mb (Doležel et al., 2003).

The DNA content data for 120 individuals were subjected to standardized processing. The average value and coefficient of variation (CV) were calculated and the variance in GS variation was analyzed using SPSS 19.0 software (SPSS, Inc.) and R studio 3.6.2. We set the GSs of the individual to be a variable, calculated the Euclidean genetic distances among the 120 samples and set 4 clustering categories. Finally, the hierarchical clustering was selected to realize the analysis with R studio 3.6.2.

## 3 RESULTS

### 3.1 GSs of the four oak species

**FIGURE 2:**
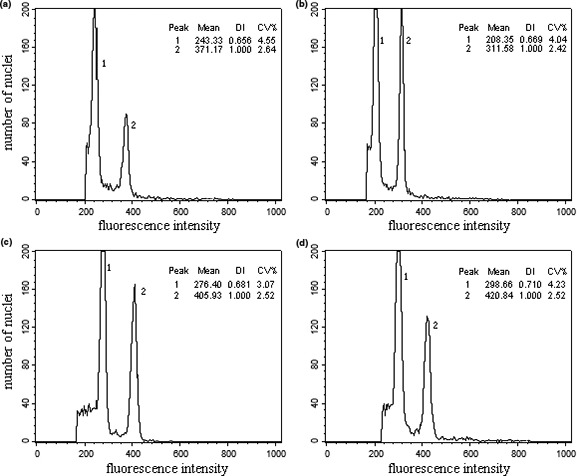
*Quercus* and *Petunia hybrida* fluorescence intensities. (a) *Q. acutissim*; (b) *Q*. *variabilis*; (c) *Q*. *Fabri*; (d). *Q*. *serrata* var. *brevipetiolata*. Peak 1: Oak nuclei at the G_0_/G_1_ phase; peak 2, *P. hybrida* nuclei at the G_0_/G_1_ phase. DI, mean nuclear DNA fluorescence index (oak/*P. hybrida*)

Owing to the low nuclear yields of mature leaves, we optimized Marie's Buffer for each of the four oak species. The CV values of the four oak species were all within the normal range, and the GSs of the individual species overlapped (Table S1). The results for the mixed samples prepared using oak and *P. hybrida* are shown in Figure 2. The oak and petunia formed narrow and high DNA peaks, respectively, and the petunia DNA peaks were generally lower than those of the oak peaks (Table 1).

**TABLE 1:** The 2C nuclear DNA contents for the four *Quercus* species measured in this study

Species	*N*	Mean 2C‐value (pg)	*SD*	MIN	MAX
*Q. acutissima*	30	1.87	0.02	1.83	1.91
*Q. variabilis*	30	1.92	0.31	1.87	1.99
*Q. fabri*	33	1.97	0.27	1.91	2.06
*Q. serrata*v ar*. brevipetiolata*	27	1.97	0.34	1.93	2.06
Average	120	1.93	0.05		

**FIGURE 3:**
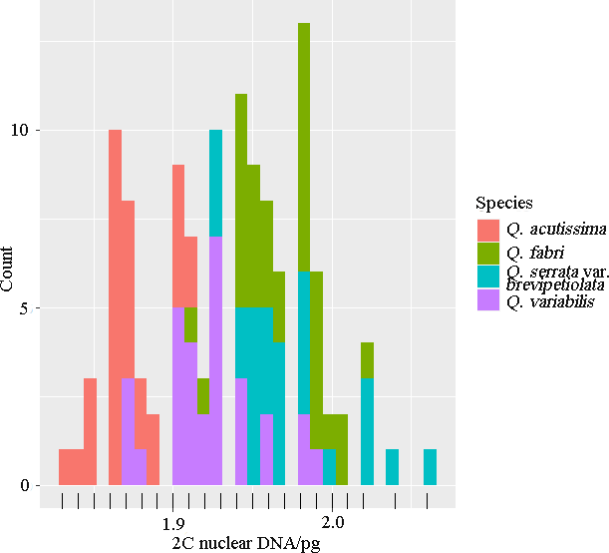
Distributions of the 2C nuclear DNA contents of 120 individuals

GS estimates varied 1.04‐fold for *Q. acutissima*, ranging from 1.83 to 1.91 pg (CV value range, 3.96–6.64), with a mean of 1.87 pg. In *Q. variabilis* individuals, estimates varied 1.06‐fold, ranging from 1.87 to 1.99 pg (CV value range, 4.26–6.78), with a mean of 1.92 pg. Estimates varied 1.05‐fold in *Q. fabri* individuals, ranging from 1.91 to 2.02 pg (CV value range, 3.11–6.71), with a mean of 1.97 pg. The GS of *Q. serrata* var. *brevipetiolata* varied 1.06‐fold, ranging from 1.93 to 2.06 pg (CV value range, 3.82–6.51), with a mean of 1.97 pg. *Quercus fabri* and *Q*. *serrata* var. *brevipetiolata* had the same mean values (1.97 pg). The average GSs for *Q. fabri* and *Q. serrata* var. *brevipetiolata* were the largest, and the average GS for *Q. variabilis* was only slightly larger than that for *Q. acutissima*. As shown in Figure 3, the GSs of most *Q. acutissima* individuals were less than the mean GS (1.82 pg), the GSs of most *Q. variabilis* individuals were greater than the mean GS (1.92 pg), and the GSs of most *Q. serrata* var. *brevipetiolata* and *Q. fabri* individuals were less than the mean GS (1.97 pg).

### 3.2 GS variation among the four oak species' populations

**FIGURE 4:**
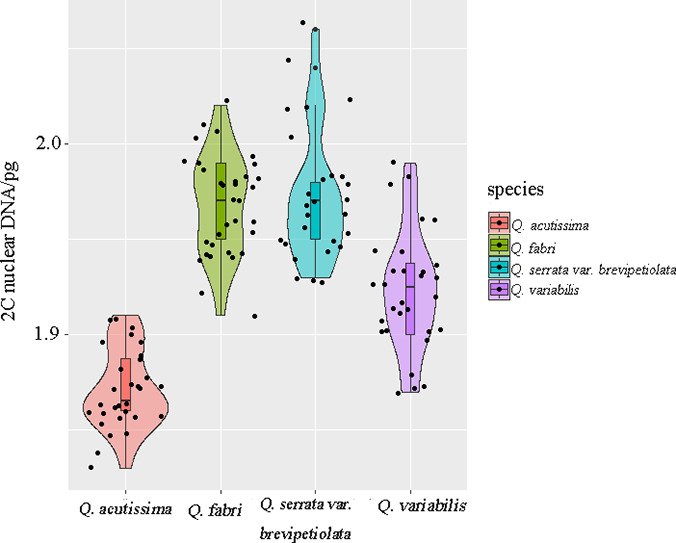
Genomic size variation in four oak species' populations

To assess the GS variation among the four oak species' populations, repeated‐measures ANOVAs were used (Table 2). There were significant differences in the GS variations among the populations, except those of *Q*. *serrata* var. *brevipetiolata* and *Q. fabri*. Additionally, variations between the populations were greater than variations within the populations. The intraspecific variation decreased in the following order: *Q*. *serrata* var. *brevipetiolata > Q. variabilis > Q. fabri* > *Q. acutissima* (Figure 4).

**TABLE 2:** An ANOVA of 2C DNA values among populations of the four *Quercus* species

pops	Source of difference		SS	*df*	MS	*F*		*p*‐value
ZJ_A × ZJ_V	Between pops	0.04		1	0.04		55.91	4.63E−10**
	Within pops	0.04		58	0.71 × 10–3			
ZJ_V × ZJ_F	Between pops	0.03		1	0.03		38.92	4.62E−08**
	Within pops	0.05		61	0.84 × 10–3			
ZJ_F × ZJ_G	Between pops	0.58 × 10^–3^		1	0.58 × 10^–3^		0.62	0.43
	Within pops	0.05		56	0.93 × 10^–3^			
ZJ_A × ZJ_F	Between pops	0.15		1	0.15		256.89	1.53E−23**
	Within pops	0.04		61	0.57 × 10^–3^			
ZJ_A × ZJ_G	Between pops	0.15		1	0.15		187.65	2.27E−19**
	Within pops	0.04		55	0.78 × 10^–3^			
ZJ_V × ZJ_G	Between pops	0.04		1	0.04		33.43	3.61E−07**
	Within pops	0.06		55	0.11 × 10^–2^			

Note

ZJ_A represents the *Q. acutissima* population, ZJ_V represents the *Q. variabilis* population, ZJ_F represents the *Q. fabri* population, and ZJ_G represents the *Q*. *serrata* var. *brevipetiolata* population.

** Statistically different (*p* < .01).

### 3.3 Clustering analysis of GS variation within and between the four *Quercus* species

**FIGURE 5:**
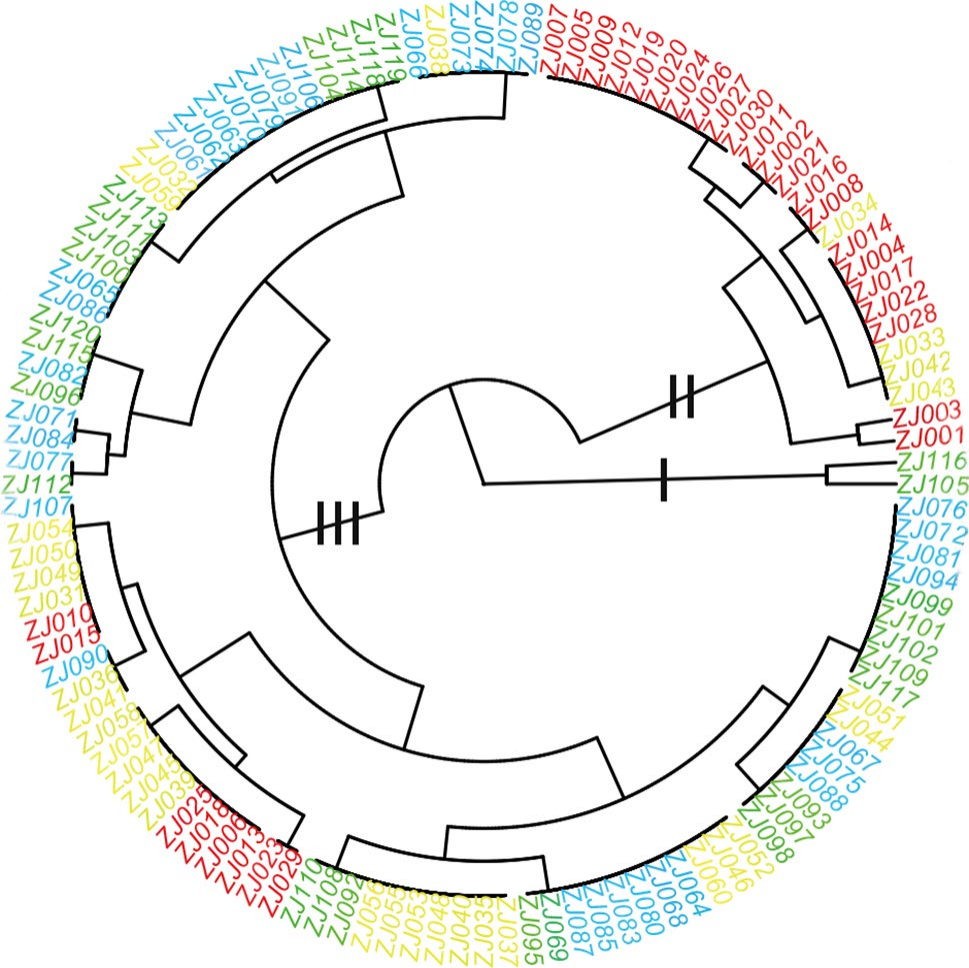
Cluster analysis results for 120 *Quercus* individuals based on nuclear 2C DNA content variation. Red, *Q. acutissima*; Yellow, *Q. variabilis*; Blue, *Q. fabri*; Green: *Q*. *serrata* var. *Brevipetiolata*

In this study, only GS variation parameters were used for cluster analysis without other indicator (Figure 5). The results showed that all the samples clustered into three clades. The two individuals ZJ116 and ZJ105 formed a single clade in the clustering analysis, and their GSs 2.024 pg and 2.06 pg, respectively, which were greater than those of the other species. This indicated that the two *Q*. *serrata* var. *brevipetiolata* individuals (ZJ116 and ZJ105) may be hybrids. The clade II was mostly composed of *Q. acutissima* individuals and a few of *Q. variabilis* individuals. The members of clade III are relatively complex, mainly including four oak species. Firstly, a small number of *Q. acutissima* individuals; secondly, *Q. variabilis* and *Q. fabri* individuals make up a large proportion of this clade, lastly the *Q*. *serrata* var. *brevipetiolata* individuals were scattered, without specific rules in this clade. The discontinuous cross‐distribution of four species of oaks in the clade III indicated that the GSs of these individuals had interspecific transition values. Moreover, *Q. acutissima* individuals did not appear in the clade which composed of *Q. fabri* and *Q*. *serrata* var. *brevipetiolata*. This confirmed that there were no interspecific hybridization between *Q. acutissima* and either *Q. fabri* or *Q*. *serrata* var. *brevipetiolata*.

### 3.4 GS variation in different evolutionary branches of angiosperms

**FIGURE 6:**
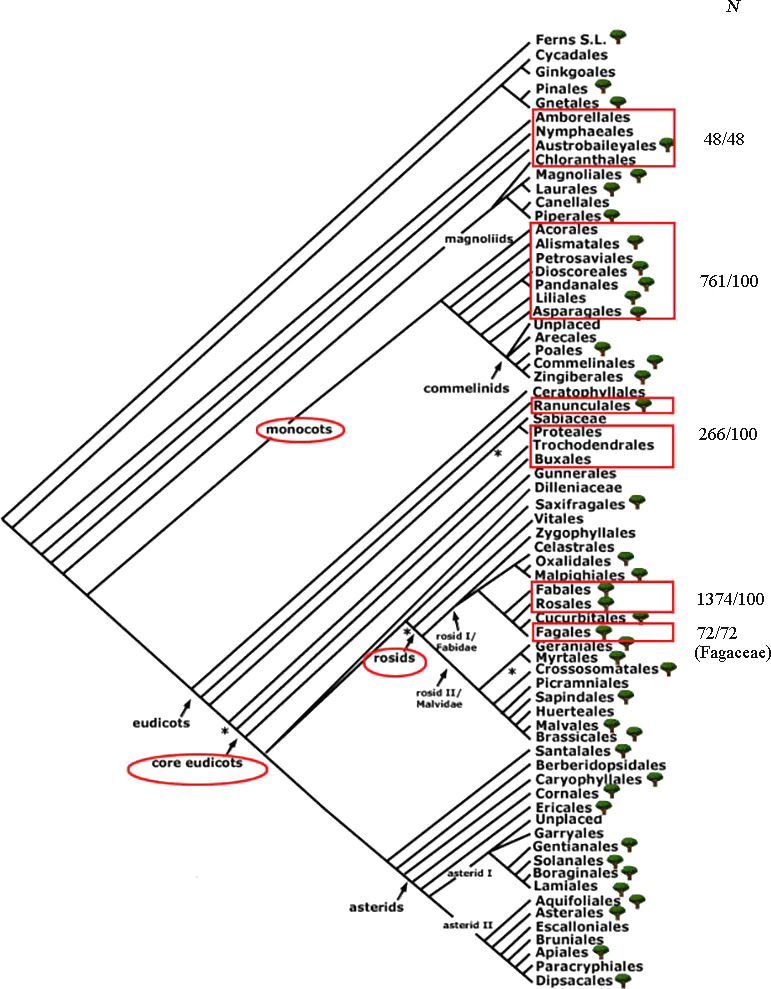
The total numbers of samples and random samples in each group. The phylogeny is adapted from Angiosperm Phylogeny Group IV

**FIGURE 7:**
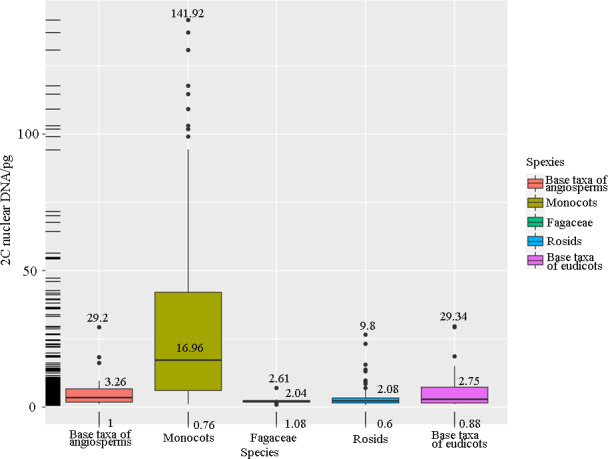
Box patterns of genomic size variation in five groups of angiosperms

It has been reported that the GSs of angiosperms are significantly non‐normal, and some lineages have extremely large GSs (Leitch & Leitch, 2013). In order to explore the characteristics of GSs of Fagaceae in angiosperms, we used the angiosperm DNA C‐value database (Leitch et al., 2019) and counted the GS variations of five groups: base taxa of angiosperm, monocots, base taxa of eudicots, rosids, and Fagaceae. Figure 6 shows the total numbers of samples and random samples in each group. The monocots had the largest variation in GS (0.76–141.92 pg). As shown in Figure 7, the mean GS of the monocots was greater than the mean of the other groups (16.96 pg). The mean GS of species in Fagaceae was small (2.04 pg).

## 4 DISCUSSION

### 4.1 The GS evolution of four oak species

The GSs in 10,770 angiosperms have been estimated and ranged between 0.062 pg (*Genlisea tuberosa*, Lentibulariaceae) and 304.46 pg (*Paris japonica*, Melanthiaceae) (Fleischmann et al., 2014; Leitch et al., 2019). The ancestral genomes of most major clades of core eudicots, such as Caryophyllales, Saxifragales, and Asterids, are very small (Soltis et al., 2003). Additionally, some genera (e.g., some Brassicaceae, Rutaceae, and Onagraceae) are clearly large genome plants. By comparing the GSs of five groups of species, we found that the GSs of Fagaceae species belong to a smaller group and the GS varies narrowly in angiosperms. This finding is consistent with that of Chen et al. (2014) who analyzed the GS variation in the Fagaceae. The Soltis division of GS was used in this study and is as follows: ≤1.4 pg, very small; >1.4 pg ≤ 3.5 pg, small; >3.5 pg ≤ 14.0 pg, intermediate; >14 pg < 35 pg, large; ≥35 pg, very large. In this study, the GS range for the four oak species was 1.83–2.06 pg. Therefore, the four oak species all had small GSs. The genomes in Sections *Quercus* and *Cerris* are about five times larger than the *Arabidopsis* genome, which has a GS that ranges from 1.84 to 2.00 pg (Aldrich & Cavender‐Bares, 2011). Our results corroborate those observed in earlier studies (Kremer et al., 2007; Leitch et al., 2019). However, the estimates for deciduous species calculated in this study were larger (1.93 vs. 1.64 pg/2C) than those calculated previously (Kremer et al., 2007). A possible explanation was that GS evolution was unidirectional, resulting in a model for overall growth (Bennetzen & Kellogg, 1997).

### 4.2 Intraspecific and interspecific variation in GS

In this study, considerable GS variability was found both within and among the four oak species. The GS estimate within the species of Section *Cerris* (1.08‐fold) was greater than that of section *Quercus* (1.07‐fold). This observation may be correlated with environmental parameters. According to the study of Zhang and Li on the prediction of the habitable zone of *Q. acutissima* and *Q. variabilis* in China, we found that optimal distribution regions for two species were the Yunnan‐Guizhou Plateau and Qin‐Ba Mountains having both a high altitude and latitude (Li et al., 2014; Zhang et al., 2014). However, the two oaks in this study came from Zijin Mountain, and their areas and altitudes are quite different compared with the most suitable areas. The Zijin Mountain is located in the plain area with an average altitude of 20–30 m, while the Yunnan‐Guizhou Plateau has an average altitude of 2,000–4,000 m, and the Qin‐Ba Mountains with altitude of 1,500–2,500 m. Species may experience natural selection when adapting to such an environment, resulting in large GS variability (Bilinski et al., 2018; Li et al., 2017). In addition, a comparison of mean GS estimates across two species in Section *Cerris* showed that the mean GS in *Q*. *acutissima* (1.87pg) is smaller than in *Q. variabilis* (1.92 pg). Some of the phenotypic traits, such as plant height, seed mass, cell size, and cell cycle time, may also facilitate GS variability (Benor et al., 2011; Kang et al., 2014; Knight et al., 2005). The differences in these phenotypic features may be related to growth rate, leaf anatomy, and photosynthesis. Previous continuous observations of the seedlings of biennial *Q. acutissima* and *Q. variabilis* revealed that the growth rate of the latter is less than that of the former (Li et al., 2020). This is in line with the theory that plans with larger genomes have lower growth rates (Kang et al., 2014; Knight et al., 2005).

The use of GS may not be very useful for classification at higher taxonomic levels, but it is particularly valuable at the species level (Liu et al., 2020; Qiu et al., 2018; Zonneveld, 2008; Zonneveld et al., 2005). The GS variation is approximately 20% across species in a single genus. In woody plants, GS and chromosomal structure are highly conserved. Therefore, the interspecific variation between genomes is greater than the intraspecies variation (Chen et al., 2014). In this study, an analysis of variance showed that most of the variation is interspecific variation. The GS variation among populations, except for those of *Q*. *serrata* var. *brevipetiolata* and *Q. fabri,* showed significant differences. Several explanations for the interspecific variation have been proposed, such as repeated cycles of polyploidy, which is supported by genomic and isozyme evidence (Bowers et al., 2003; Otto & Whitton, 2000; Wendel, 2000). Earlier studies indicated that the interspecific variation in GS results partly from the appearance of extra B chromosomes, which are caused by the irregular segregation of additional chromosomes during mitosis (Piscor & Parise‐Maltempi, 2015; Zoldos et al., 1998). However, recent technological advances have shown that the presence of B chromosomes generally increases the size of an individual genome, but it does not affect the extent of the variation within the population, regardless of whether it includes individuals having satellite chromosomes. The new findings also suggest that the GS variation may be related to the A chromosome (Chumová et al., 2016). These hypotheses are all possible because the chromosome and ploidy numbers in the Fagaceae remain stable (2n = 24) in most genera, except for extra chromosomes in some *Quercus* populations (Dzialuk et al., 2007; Zoldos et al., 1998). When the number of chromosome changes results from fission and fusion, then the evolution of the chromosomes may result in recombination between populations. Here, we speculated that the variation in GS was owing to hybridization. We suspect that ZJ116 and ZJ105 were hybrid individuals using a clustering analysis with GS expansion (2.024 pg and 2.06 pg). They may be hybrids resulting from crosses between two species in Section *Quercus*. The white oaks are wind‐pollinated and unable to discriminate pollen from other species in the same section. In addition, we speculated that hybrid offspring having expanded GSs were produced from Section *Quercus* and Section *Cerris*. Cluster analysis showed that there were always *Q. fabri* and *Q. serrata* var. *brevipetiolata* individuals interspersed in the *Q. variabilis* population. The phenomenon of hybridization within different groups is rare and blocked by reproductive isolation, but it exists and has been reported (Burgarella et al., 2009). In general, a GS increases after polyploidization, but it may undergo a decrease in noncoding DNA sequences, leading to a reduced GS after polyploidization (Li et al., 2013). Furthermore, an increase or decrease in DNA repeat sequences during oak hybridization leads to variations in GS and is the main reason for GS changes in angiosperms.

### 4.3 Ecological protection proposals for oak trees

Interspecific hybridization and the introgression of *Quercus* leads to a series of systematic evolutionary and ecological results, such as community recombination and structural adjustment (Aldrich & Cavender‐Bares, 2011; McVay et al., 2017; Song et al., 2015). In addition to the impact on community succession, the hybridization and introgression of oaks are conducive to increasing the genetic diversity and the rapid transformation and fixation of adaptive genes among species. This is conducive to the better survival of species in new environments (Ramirez‐Valiente & Cavender‐Bares, 2017; Ramirez‐Valiente et al., 2018). However, closely related species seldom coexist owing to functional divergences that allow them to occupy different habitats (Klein et al., 2016). In our study, the four oak species were found coexisting on the same land. Their coexistence may result from the vegetation in Zijin Mountains being mainly artificial and natural secondary forests. In the 1950s and 1960s, the inbreeding decline of related species was not considered in afforestation. The GS variations in the four species of *Quercus* may have been required to adapt to such an environment. In addition, the hybridization in different sections may have resulted from habitat disturbance. As a tourist attraction, Zijinshan has year‐round human activities that change the diffusion patterns of seeds and pollens, disturbing the habitat to a certain extent. Thus, local authorities should guide tourists, strengthen the ecological management, perform appropriate thinning activities, and reduce inbreeding.

## 5 CONCLUSIONS

In this study, the mean GSs of *Q. acutissima*, *Q. variabilis*, *Q. fabri*, and *Q*. *serrata* var. *brevipetiolata* were 1.87, 1.92, 1.97, and 1.97 pg, respectively, which were within a reasonable range. Thus, there was a low level of intraspecific variation in GSs among *Q. acutissima*, but it was relatively high among individuals of the other three oak species. Furthermore, there was a high level of interspecific variation among the four oak populations. The oaks in the same section produced hybrid introgression. Additionally, a hybrid offspring was produced from *Q. fabri* and *Q. variabilis*, which belong to different sections. The pattern of GS evolution for hybrids species is expansion. This study on GS described a valuable complementary method for studying genetic variation in oak species and has significance in guiding the ecological protection of oaks.

## CONFLICT OF INTEREST

None declared.

## AUTHOR CONTRIBUTIONS

**GaoMing Wei:** Data curation (lead); formal analysis (lead); methodology (lead); resources (equal); writing–original draft (equal). **Xuan Li:** Formal analysis (supporting); methodology (supporting); software (lead); writing–original draft (equal); writing–review and editing (lead). **YanMing Fang:** Resources (equal); supervision (lead); validation (equal); writing–review and editing (supporting).

## Supporting information

Table S1

## Data Availability

Genome size (GS) database for 120 individuals of four *Querucs* species is publicly available at Dryad repository: https://doi.org/10.5061/dryad.hx3ffbgcv.
