# Supplementary material for: Sympatric genome size variation and hybridization of four oak species as determined by flow cytometry genome size variation and hybridization
Source: Ecol Evol. 2021 Jan 14;11(4):1729–40. doi: 10.1002/ece3.7163 (PMC7882991; doi:10.1002/ece3.7163)
Supplement: Supplementary file 1 — Table S1 [file ECE3-11-1729-s001.docx]

Appendix

**Table S1** GSs of 120 *Quercus* sp. individuals

| Species | Sample number | DI | SD | 2C nuclear DNA/pg | 2C nuclear DNA/Mbp | CV% |
| --- | --- | --- | --- | --- | --- | --- |
|  | ZJ001 | 0.643 | 0.013 | 1.83 | 1793 | 6.32 |
|  | ZJ002 | 0.650 | 0.003 | 1.85 | 1812 | 5.75 |
|  | ZJ003 | 0.646 | 0.003 | 1.84 | 1800 | 6.11 |
|  | ZJ004 | 0.656 | 0.001 | 1.87 | 1827 | 6.00 |
|  | ZJ005 | 0.654 | 0.001 | 1.86 | 1822 | 5.36 |
|  | ZJ006 | 0.665 | 0.009 | 1.90 | 1853 | 6.64 |
|  | ZJ007 | 0.652 | 0.002 | 1.86 | 1818 | 5.26 |
|  | ZJ008 | 0.659 | 0.002 | 1.88 | 1836 | 3.96 |
|  | ZJ009 | 0.652 | 0.003 | 1.86 | 1818 | 5.63 |
|  | ZJ010 | 0.669 | 0.001 | 1.91 | 1863 | 5.04 |
|  | ZJ011 | 0.650 | 0.002 | 1.85 | 1812 | 5.83 |
|  | ZJ012 | 0.653 | 0.001 | 1.86 | 1821 | 4.52 |
|  | ZJ013 | 0.665 | 0.002 | 1.90 | 1855 | 6.13 |
|  | ZJ014 | 0.655 | 0.002 | 1.87 | 1826 | 5.26 |
| *Q. acutissima* | ZJ015 | 0.671 | 0.004 | 1.91 | 1870 | 4.90 |
|  | ZJ016 | 0.658 | 0.002 | 1.88 | 1835 | 6.43 |
|  | ZJ017 | 0.654 | 0.002 | 1.87 | 1824 | 4.65 |
|  | ZJ018 | 0.665 | 0.001 | 1.90 | 1854 | 5.17 |
|  | ZJ019 | 0.654 | 0.001 | 1.86 | 1824 | 4.87 |
|  | ZJ020 | 0.654 | 0.005 | 1.86 | 1822 | 5.82 |
|  | ZJ021 | 0.649 | 0.003 | 1.85 | 1808 | 5.91 |
|  | ZJ022 | 0.656 | 0.005 | 1.87 | 1828 | 6.38 |
|  | ZJ023 | 0.663 | 0.002 | 1.89 | 1847 | 5.35 |
|  | ZJ024 | 0.653 | 0.003 | 1.86 | 1819 | 5.63 |
|  | ZJ025 | 0.667 | 0.004 | 1.90 | 1859 | 5.94 |
|  | ZJ026 | 0.652 | 0.001 | 1.86 | 1818 | 4.70 |
|  | ZJ027 | 0.652 | 0.002 | 1.86 | 1817 | 5.16 |
|  | ZJ028 | 0.655 | 0.003 | 1.87 | 1827 | 5.55 |
|  | ZJ029 | 0.662 | 0.001 | 1.89 | 1845 | 4.49 |
|  | ZJ030 | 0.652 | 0.005 | 1.86 | 1818 | 5.95 |
|  | ZJ031 | 0.670 | 0.002 | 1.91 | 1866 | 6.18 |
|  | ZJ032 | 0.695 | 0.002 | 1.98 | 1937 | 5.81 |
|  | ZJ033 | 0.656 | 0.001 | 1.87 | 1830 | 4.71 |
|  | ZJ034 | 0.659 | 0.004 | 1.88 | 1838 | 4.74 |
|  | ZJ035 | 0.679 | 0.002 | 1.93 | 1892 | 5.80 |
|  | ZJ036 | 0.675 | 0.001 | 1.92 | 1882 | 5.81 |
|  | ZJ037 | 0.678 | 0.001 | 1.93 | 1889 | 6.06 |
|  | ZJ038 | 0.699 | 0.001 | 1.99 | 1949 | 6.15 |
|  | ZJ039 | 0.667 | 0.002 | 1.90 | 1858 | 6.78 |
|  | ZJ040 | 0.678 | 0.004 | 1.93 | 1890 | 6.43 |
|  | ZJ041 | 0.674 | 0.001 | 1.92 | 1880 | 6.00 |
| *Q. variabilis* | ZJ042 | 0.657 | 0.002 | 1.87 | 1831 | 5.96 |
|  | ZJ043 | 0.657 | 0.001 | 1.87 | 1832 | 6.11 |
|  | ZJ044 | 0.689 | 0.001 | 1.96 | 1920 | 6.20 |
|  | ZJ045 | 0.666 | 0.003 | 1.90 | 1857 | 4.26 |
|  | ZJ046 | 0.681 | 0.002 | 1.94 | 1898 | 5.50 |
|  | ZJ047 | 0.665 | 0.004 | 1.90 | 1853 | 6.78 |
|  | ZJ048 | 0.679 | 0.002 | 1.93 | 1891 | 6.23 |
|  | ZJ049 | 0.670 | 0.001 | 1.91 | 1867 | 6.25 |
|  | ZJ050 | 0.670 | 0.002 | 1.91 | 1866 | 5.23 |
|  | ZJ051 | 0.688 | 0.002 | 1.96 | 1918 | 5.59 |
|  | ZJ052 | 0.679 | 0.004 | 1.94 | 1893 | 5.82 |
|  | ZJ053 | 0.677 | 0.002 | 1.93 | 1888 | 5.05 |
|  | ZJ054 | 0.669 | 0.003 | 1.91 | 1865 | 6.39 |
|  | ZJ055 | 0.678 | 0.002 | 1.93 | 1889 | 4.47 |
|  | ZJ056 | 0.677 | 0.003 | 1.93 | 1887 | 5.95 |
|  | ZJ057 | 0.666 | 0.001 | 1.90 | 1856 | 5.31 |
|  | ZJ058 | 0.668 | 0.004 | 1.90 | 1862 | 5.79 |
|  | ZJ059 | 0.693 | 0.001 | 1.98 | 1933 | 6.22 |
|  | ZJ060 | 0.680 | 0.001 | 1.94 | 1896 | 5.55 |
|  | ZJ061 | 0.694 | 0.000 | 1.98 | 1936 | 3.96 |
|  | ZJ062 | 0.695 | 0.001 | 1.98 | 1937 | 5.01 |
|  | ZJ063 | 0.696 | 0.002 | 1.98 | 1940 | 6.71 |
|  | ZJ064 | 0.682 | 0.000 | 1.94 | 1902 | 5.36 |
|  | ZJ065 | 0.692 | 0.001 | 1.97 | 1929 | 4.44 |
|  | ZJ066 | 0.698 | 0.001 | 1.99 | 1946 | 4.87 |
|  | ZJ067 | 0.687 | 0.005 | 1.96 | 1915 | 5.49 |
|  | ZJ068 | 0.680 | 0.000 | 1.94 | 1896 | 3.11 |
|  | ZJ070 | 0.694 | 0.001 | 1.98 | 1933 | 5.53 |
|  | ZJ071 | 0.705 | 0.000 | 2.01 | 1964 | 5.71 |
|  | ZJ072 | 0.684 | 0.004 | 1.95 | 1907 | 5.87 |
|  | ZJ073 | 0.697 | 0.001 | 1.99 | 1943 | 4.36 |
|  | ZJ074 | 0.697 | 0.002 | 1.99 | 1942 | 5.16 |
|  | ZJ075 | 0.686 | 0.000 | 1.96 | 1913 | 3.97 |
| *Q. fabri* | ZJ076 | 0.685 | 0.001 | 1.95 | 1910 | 5.71 |
|  | ZJ077 | 0.700 | 0.000 | 2.00 | 1951 | 5.42 |
|  | ZJ078 | 0.699 | 0.001 | 1.99 | 1948 | 4.16 |
|  | ZJ079 | 0.695 | 0.001 | 1.98 | 1938 | 4.56 |
|  | ZJ080 | 0.679 | 0.001 | 1.94 | 1894 | 5.23 |
|  | ZJ081 | 0.684 | 0.001 | 1.95 | 1908 | 4.11 |
|  | ZJ082 | 0.709 | 0.001 | 2.02 | 1976 | 3.34 |
|  | ZJ083 | 0.679 | 0.001 | 1.94 | 1893 | 5.17 |
|  | ZJ084 | 0.707 | 0.002 | 2.01 | 1971 | 4.87 |
|  | ZJ085 | 0.681 | 0.001 | 1.94 | 1898 | 5.28 |
|  | ZJ086 | 0.692 | 0.002 | 1.97 | 1928 | 3.43 |
|  | ZJ087 | 0.682 | 0.005 | 1.94 | 1900 | 5.52 |
|  | ZJ088 | 0.689 | 0.000 | 1.96 | 1922 | 3.42 |
|  | ZJ089 | 0.699 | 0.000 | 1.99 | 1949 | 4.57 |
|  | ZJ090 | 0.674 | 0.002 | 1.92 | 1878 | 4.65 |
|  | ZJ091 | 0.694 | 0.003 | 1.98 | 1933 | 5.83 |
|  | ZJ094 | 0.685 | 0.001 | 1.95 | 1909 | 4.51 |
|  | ZJ106 | 0.696 | 0.001 | 1.98 | 1940 | 5.64 |
|  | ZJ107 | 0.671 | 0.002 | 1.91 | 1871 | 5.92 |
|  | ZJ069 | 0.681 | 0.000 | 1.94 | 1898 | 4.01 |
|  | ZJ092 | 0.676 | 0.001 | 1.93 | 1884 | 4.26 |
|  | ZJ093 | 0.689 | 0.001 | 1.96 | 1921 | 4.81 |
|  | ZJ095 | 0.682 | 0.001 | 1.94 | 1900 | 3.82 |
|  | ZJ096 | 0.707 | 0.009 | 2.02 | 1971 | 5.36 |
|  | ZJ097 | 0.686 | 0.002 | 1.96 | 1912 | 5.11 |
|  | ZJ098 | 0.689 | 0.003 | 1.96 | 1921 | 6.51 |
|  | ZJ099 | 0.685 | 0.001 | 1.95 | 1909 | 4.21 |
|  | ZJ100 | 0.692 | 0.003 | 1.97 | 1930 | 5.39 |
|  | ZJ101 | 0.685 | 0.003 | 1.95 | 1910 | 4.91 |
| *Q. glandulifera*  var*. brevipetiolata* | ZJ102 | 0.684 | 0.002 | 1.95 | 1906 | 5.74 |
| var*. brevipetiolata* | ZJ103 | 0.692 | 0.000 | 1.97 | 1928 | 5.00 |
|  | ZJ104 | 0.695 | 0.001 | 1.98 | 1937 | 5.56 |
|  | ZJ105 | 0.722 | 0.002 | 2.06 | 2012 | 5.68 |
|  | ZJ108 | 0.677 | 0.001 | 1.93 | 1886 | 5.81 |
|  | ZJ109 | 0.685 | 0.003 | 1.95 | 1909 | 5.88 |
|  | ZJ110 | 0.678 | 0.002 | 1.93 | 1890 | 5.20 |
|  | ZJ111 | 0.691 | 0.003 | 1.97 | 1926 | 5.52 |
|  | ZJ112 | 0.702 | 0.002 | 2.00 | 1956 | 4.50 |
|  | ZJ113 | 0.690 | 0.001 | 1.97 | 1923 | 5.26 |
|  | ZJ114 | 0.693 | 0.001 | 1.98 | 1932 | 4.71 |
|  | ZJ115 | 0.708 | 0.001 | 2.02 | 1973 | 5.26 |
|  | ZJ116 | 0.714 | 0.000 | 2.04 | 1991 | 5.28 |
|  | ZJ117 | 0.685 | 0.001 | 1.95 | 1909 | 4.27 |
|  | ZJ118 | 0.695 | 0.000 | 1.98 | 1937 | 5.15 |
|  | ZJ119 | 0.693 | 0.001 | 1.98 | 1932 | 5.79 |
|  | ZJ120 | 0.710 | 0.002 | 2.02 | 1980 | 4.09 |

DI, the mean nuclear DNA fluorescence index (oak/*P. hybrida*); SD, standard deviation; CV, the mean coefficient of variation.
